# Telemonitoring Potential of Wearable Cardioverter-Defibrillators during the Follow-Up of Patients with Heart Failure

**DOI:** 10.3390/jcdd9060175

**Published:** 2022-06-01

**Authors:** Christian Blockhaus, Jan-Erik Guelker, Ludger Feyen, Alexander Bufe, Melchior Seyfarth, Dong-In Shin

**Affiliations:** 1 Heart Centre Niederrhein, Department of Cardiology, Helios Clinic Krefeld, 47805 Krefeld, Germany; alexander.bufe@helios-gesundheit.de (A.B.); dong-in.shin@helios-gesundheit.de (D.-I.S.); 2 Faculty of Health, School of Medicine, University Witten/Herdecke, 58448 Witten, Germany; janguelker@gmx.de (J.-E.G.); ludger.feyen@helios-gesundheit.de (L.F.); melchior.seyfarth@helios-gesundheit.de (M.S.); 3 Department of Cardiology, Petrus Hospital, 42283 Wuppertal, Germany; 4 Department of Diagnostic and Interventional Radiology, Helios Clinic Krefeld, 47805 Krefeld, Germany; 5 Department of Diagnostic and Interventional Radiology, Helios University Hospital, 42283 Wuppertal, Germany; 6 Department of Cardiology, Helios University Hospital, 42283 Wuppertal, Germany

**Keywords:** wearable cardioverter defibrillator, sudden cardiac death, heart failure unit, telemonitoring, physical activity

## Abstract

Background: Wearable cardioverter-defibrillators (WCDs) are a well-established tool to bridge the recovery time of left ventricular ejection fraction (LVEF) until the implantation of an implantable cardioverter defibrillator (ICD), as recommended by the current guidelines. Besides their function to detect and treat malignant arrhythmias, WCDs may be used as a telemonitoring system. In this study, we sought to illustrate and discuss the telemonitoring potential of WCDs and to analyze physical activity in specific patient cohorts. Methods and Results: We retrospectively included 140 patients with reduced LVEF who were prescribed WCDs in our clinic. We analyzed the patients’ physical activity (*n* = 105 with a WCD compliance above 21 h/day), body position and resting position. We found a reduced physical activity in women and in patients over the age of 65 compared to younger patients. Furthermore, the patients who were overweight or obese showed significantly reduced physical activity compared to the patients with a normal weight (6365 ± 3572 vs. 4972 ± 2476 vs. 7045 ± 3521, *p* = 0.02). Conclusion: WCDs may be used as a telemonitoring and intervention tool in patients with reduced LVEF. Specific patient groups may benefit from guidance from their treating physician regarding physical activity.

## 1. Introduction

Patients with reduced left ventricular ejection fraction (LVEF) are exposed to a higher risk of ventricular arrhythmias and sudden cardiac death (SCD). The main causes of reduced LVEF and consecutive heart failure (HF) are ischemic cardiomyopathy (ICM) based on coronary artery disease (CAD) and dilated cardiomyopathy (DCM) [1]. The global burden of HF with reduced LVEF (HFrEF) has increased significantly over the last few decades [2]. Besides the implementation of optimal HF therapy, the implantation of either an implantable cardioverter defibrillator (ICD) or a cardiac resynchronization therapy defibrillator (CRT-D) has become an option for the primary or secondary prevention of SCD. The current guidelines recommend the implantation of a device after a specific time of optimal medical therapy in order to allow LVEF to recover. During this period, wearable cardioverter-defibrillators (WCDs) may serve as a bridge to the recovery of LVEF or as a bridge to implantation and are recommended for patients with a specific risk of SCD [3]. This recommendation is mostly based on registry studies and one randomized study [4,5]. The same guidelines also recommend exercise for stable patients and the use of telemonitoring systems. It is known that patients with HF and reduced physical activity (e.g., measured by the number of daily steps) have a worse outcome than physically active patients. Furthermore, changes in body angle and body position may be precursors to progressing HF. 

Besides its function to detect and treat malignant arrhythmias, WCDs may be used as telemonitors to track patients’ compliance, daily steps and heart rate, along with their body position, body angle and position during rest. The aim of this study was to illustrate and discuss the telemonitoring potential of WCDs in HFrEF patients during the time of WCD prescription in a retrospective cohort.

## 2. Materials and Methods

The WCD used (LifeVest^®^ 4000 Model (LifeVest system, ZOLL, Pittsburgh, PA, USA)) consists of an electrode belt with non-adhesive electrodes and self-gelling defibrillation electrodes in the posterior and lateral positions. In case of the detection of ventricular tachycardia (VT) or ventricular fibrillation (VF), the device generates several alarms before a biphasic shock of up to 150 Joule is delivered. The shock delivery can be prevented by pushing the response button. For telemonitoring purposes, the online platform “LifeVest^®^ network” is provided by the manufacturer and can be accessed via a password secured by the prescribing physician. The system automatically tracks all potentially life-threatening arrhythmias, interventions (i.e., alarms and shock delivery) and continuous information such as the heart rate, level of physical activity and wearing compliance counted in terms of hours per day (h/d). As for telemonitoring, the supplier’s online platform (“LifeVest^®^ network”) can be accessed by the treating physician. The system records life-threatening arrhythmias (and non-sustained VT), interventions and continuous data such as heartrate, level of physical activity and wearing, body position and the patient’s compliance.

We retrospectively screened a cohort of 200 patients who received a WCD in our clinic between 2016 and 2020. The partial analysis of this patient collective has been previously published elsewhere [6]. Here, we analyzed 140 patients with an initial diagnosis of ICM or DCM. All of the patients had been presented to our hospital for elective or emergency treatment. All of the patients were treated according to the current guidelines. The patient’s medical history was analyzed with respect to comorbidities (e.g., CAD, arterial hypertension, history of stroke, atrial fibrillation, diabetes mellitus type II, obstructive sleep apnea syndrome or chronic obstructive pulmonary disease), medication at discharge, body mass index (BMI), creatinine (mg/dL) and initial brain natriuretic peptide (BNP, pg/mL). The initial LVEF was documented either by transthoracic echocardiography (TTE) or by magnetic resonance imaging (MRI). All of the patients where offered a three-month follow-up in our outpatient’s clinic, or the follow-up was undertaken by an outpatient’s cardiologist to reevaluate the LVEF and to screen for the indication of the implantation of an ICD/CRT-D. All of the follow-up data recorded by the WCD were retrospectively accessed via the online platform of the WCD supplier. In this study, we analyzed patients’ daily step count, heart rate, average body position and resting position.

**Figure 1:**
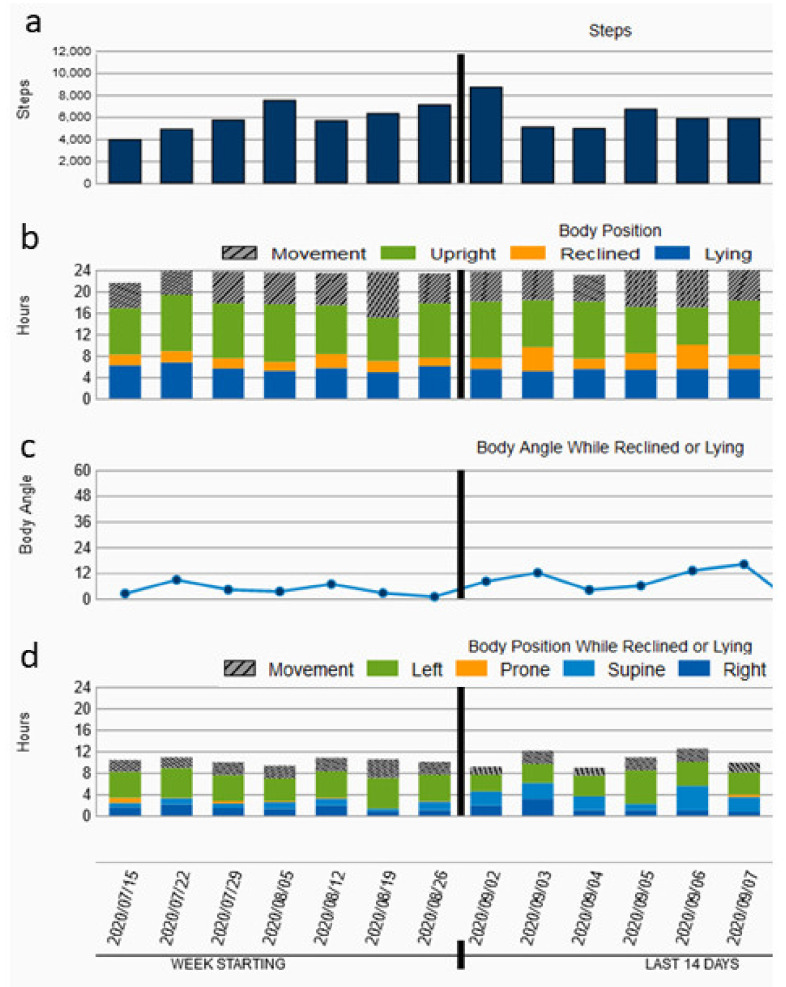
The patient’s (**a**) daily steps, (**b**) body position, (**c**) body angle while reclined or lying and (**d**) body position while reclined or lying over the time of the WCD prescription.

Figure 1 illustrates an example of a patient’s tracked steps per day (Figure 1a), body position (Figure 1b), body angle while reclined or lying (Figure 1c) and body position while reclined or lying (Figure 1d).

The study design was approved by the ethics committee of Ärztekammer Nordrhein (2020014) and conformed to the standards defined in the Helsinki Declaration.

Statistical analysis: The continuous data are shown as the mean ± standard deviation (SD). Fisher’s exact test was used to test for the independence of the two categorical variables. The means of the continuous data of the independent groups were compared by a *t*-test (two groups) or *F*-test (three groups). In addition, linear regression was used as a multivariate approach to estimate the effects of BMI and age on daily steps and the difference between men and women. All of the statistical tests were two-sided at a significance level of 0.05. The statistical analysis was performed using Stata/IC 16.1 for Unix (StataCorp, LLC, College Station, TX, USA).

## 3. Results

We analyzed 140 patients (32 (22.86%) females) with a mean age of 57.91 ± 12.76 years who received a WCD between 2016 and 2020 in our clinic. In 78 (55.71%) cases, the patient’s indication was ICM; in 62 (44.29%) cases, the indication was DCM. The diagnosis was documented the first time in 104 (74.28%) of the cases. The mean initial LVEF in all of the patients was 26.9% ± 7.35, with a mean initial BNP of 4798 ± 6769 pg/mL. The average New-York-Hear-Association (NYHA I-IV) stage was 2.80 ± 0.86. The initial baseline characteristics can be found in Table 1.

At discharge, most of the patients were treated according to the current guidelines. In total, 140 (100%) of the patients were treated with betablockers, 99 (70.71%) were treated with angiotensin-converting enzyme inhibitors (ACE-I) or angiotensin receptor antagonists (AT), 127 (90.71%) were treated with aldosterone receptor antagonists and 40 (28.57%) were treated with an AT/neprilysin inhibitor. A total of 53 (37.85%) patients received anticoagulant therapy. The medication at discharge is shown in Table 2.

The patients were instructed to wear the WCD as often as possible and with the fewest possible interruptions. After a mean WCD use of 59.78 ± 35.72 days, the overall compliance was 21.39 ± 3.98 h/d. The mean LVEF increased to 37.05 ± 9.12%. In 39 (27.85%) cases, an ICD or CRT-D was implanted. The analyzed follow-up data recorded by the WCD are listed in Table 3.

As ivabradine is only recommended and used in patients with sinus rhythm who are presenting with a persistent heart rate above 70 bpm, we analyzed the mean heart rate for this cohort during the time of WCD prescription, consisting of 100 (57.8%) patients with an overall mean heart rate of 69.92 ± 10.96 bpm. During the follow-up, in five (3.57%) patients, VT was documented by the WCD with one adequate shock delivery. The other VTs were either self-limiting or the patient pushed the response button. All of these five patients were male, with a mean age of 57.8 ± 4.15 years and a good compliance of 23.52 ± 0.28 h/d.

**Figure 2:**
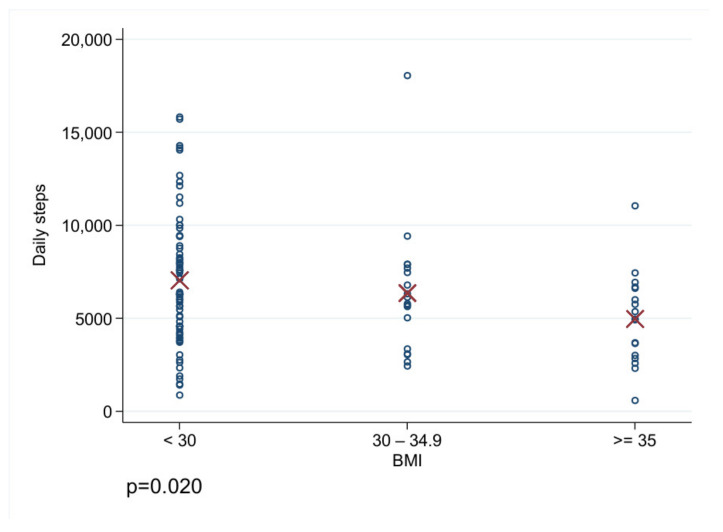
Daily steps of the three groups: 1 = BMI <30, 2 = BMI 30–34.9, 3 = BMI > = 35.

**Figure 3:**
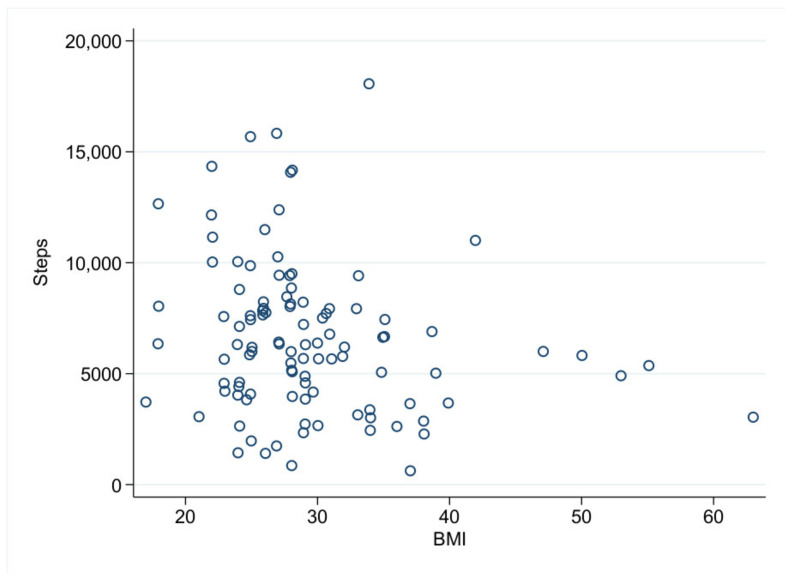
Steps vs. BMI (Scatterplot).

With regard to the analysis of the daily step count, we analyzed 105 (60.69%) patients with a compliance of more than 21 h/d. Here, we found a mean of 6593 ± 3440 steps per day. In this specific cohort, we found significant differences concerning the daily steps with regard to the patients’ age and BMI. Furthermore, the males tended to walk more steps per day than the females (6966 ± 3571 vs. 5661 ± 2940, *p* = 0.058). The patients younger than 65 years walked 7070 ± 3618 steps per day, whereas the patients ≥65 years only walked 5714 ± 2931 steps per day (*p* = 0.04). With regard to BMI, the patients with a BMI <30 walked 7044 ± 3521 steps per day, the patients with a BMI between 30 and 34.9 walked 6365 ± 3571 steps per day and the patients with a BMI ≥35 walked 4971 ± 2476 steps per day (*p* = 0.02, Figure 2). In total, there is significant dependency between BMI and age (*p* = 0.006), and the elderly patients showed a BMI below 30 more often than the younger patients did. The multivariate analysis revealed age, BMI and sex as independent factors with a significant influence on daily steps (Table 4). The men walked more than the women, and an increased BMI and increased age led to fewer daily steps. Each additional BMI point is associated with 117 fewer steps per day; the negative correlation between daily steps and BMI is illustrated in Figure 3.

Furthermore, as shown in Table 3, we analyzed the patients’ average body position. The patients’ position was mostly upright (52.15 ± 15.67%), followed by a reclined position (17.64 ± 10.51%) and a flat position (30.15 ± 14.93%). Furthermore, the sleeping position was analyzed, showing a left position in 22.59 ± 14.53%, a prone position in 9.31 ± 13.38, a right position in 21.23 ± 14.58% and a supine position in 46.91 ± 20.45%.

## 4. Discussion

Our study illustrates the potential of WCDs to be used as a telemonitoring system in patients with congestive heart failure during the period of WCD prescription until a decision is made concerning ICD or CRT-D implantation. Furthermore, the study shows that older patients and patients who are overweight or obese walk significantly fewer steps per day than younger patients and patients with a normal weight do. As we only had retrospective access to the telemonitoring system, these findings had no impact on the treatment of our patients.

The new guidelines for diagnosing and treating acute and chronic heart failure, which were published in 2021, recommend WCDs for selected patients with an increased risk of SCD. The recommendation consists of a Class IIb indication [3]. WCDs are mainly seen as a bridge to LVEF restoration or as a strategy to be employed until the implantation of an ICD or CRT-D system. The above-mentioned guidelines also recommend the use of home telemonitoring systems with a Class IIb indication, with the aim of reducing cardiovascular death and hospitalization due to heart failure. Furthermore, exercise is recommended with a Class Ia indication. Patients should be advised to exercise while recognizing limitations such as comorbidities, frailty, etc. 

The 2021 European guidelines on cardiovascular disease prevention in clinical practice recommend physical activity of moderate intensity for 150–300 min per week or physical activity of vigorous intensity for 75–150 min per week for persons of all ages (Class Ia recommendation). Patients with heart failure who are symptomatically stable should be offered an exercise-based cardiac rehabilitation (Class Ia recommendation) [7]. The same goals concerning weekly physical activity are defined by the physical activity guidelines for Americans published in 2018. Here, 150–300 min of physical activity per week is also advised for adults with chronic disabilities or conditions [8]. In 2020, Saint-Maurice et al., published data on 4840 patients which showed that a greater number of daily steps was significantly associated with a lower all-cause mortality. In detail, they found significant differences between the patients walking 4000, 8000 or 12,000 steps per day [9]. Burch et al., reported in the WCD trial a reduced activity prior to ventricular arrhythmias in WCD patients [10]. Tripp et al., found that physical activity measured by daily steps with the WCD was modest after hospital discharge and increased during the time of WCD prescription in patients after myocardial infarction. Nevertheless, the patients’ overall mean daily steps rate was only 5568 [11]. In our cohort, the daily steps for all of the patients were comparable with 6593 ± 3564. We found significantly fewer daily steps walked by females than those walked by males and fewer daily steps walked by patients older than 65 compared to those walked by younger patients. It is worth mentioning that we usually do not prescribe WCDs to patients who are not mobile or physically active at all or who are significantly older than 80 years—except for rare cases. Of note, the WCD cannot detect stationary physical activity, but the daily step counter seems to be a good parameter to analyze daily activity as long as the WCD is worn for most of the day. Concerning the daily compliance, it is known that elderly patients and women show a better adherence than younger and male patients [6,12]. Thus, WCDs may serve as a telemonitoring system to supervise, remind and train patients or to motivate them to maintain or augment physical activity (Figure 1a). On the other hand, when the daily steps decrease during observation, this could be an alarm signal indicating the worsening of HF. It is known that patients with higher NYHA classifications are associated with less physical activity [13]. The patients who are overweight or obese exhibited significantly reduced physical activity compared to the patients with a normal weight (Figure 2). 

In our cohort, sex, age and BMI were independent factors with regard to daily steps. However, due to the retrospective character of this study, we cannot provide data regarding whether these findings had an impact on the patients’ outcomes.

It is known that women have a more favorable outcome in heart failure than men do, even when presenting with more comorbidities [14,15,16]. Women are often underrepresented in many studies, and this is true of our study. It is debatable why we found women to show less physical activity than men. This may be due to the aforementioned underrepresentation or to an avoidance strategy while wearing WCDs.

WCDs may also be used as a monitor of sleeping position and changes in one’s preferred position (Figure 1d). It is known that adults mostly prefer the right-sided sleeping position (RSSP) [17]. Furthermore, people with CHF seem to prefer the RSSP compared to the left-sided sleeping position (LSSP) [18,19]. Bayraktar et al., reported in their echocardiographic study that patients with heart failure show significantly higher TAPSE and LVOT TVI parameters in the RSSP than patients in the LSSP [20]. Joho et al., reported on the influence of sleeping position in patients with heart failure and central sleep apnea. They found that positional therapy decreased sleep apnea syndrome and BNP levels significantly [21]. Thus, WCDs may indicate changes in sleeping positions due to the worsening of heart failure. Additionally, WCDs may track the body angle during sleep (Figure 1c). The changes here may also be indicators of changes in heart failure severity, as patients with severe heart failure tend to sleep in a more semi-recumbent position [22,23]. Notably, in this retrospective study, we cannot correlate the patients’ outcome with the analyzed body positions and angles. Here, further prospective and randomized studies should be conducted.

Our study aimed at illustrating and discussing the potential of WCDs in telemonitoring HFrEF patients during the time of WCD prescription. Furthermore, we characterized specific cohorts of patients who show significantly reduced physical activity and who may benefit from interventions by their treating heart failure specialist. Heart failure units and heart failure experts may use this potential to monitor, supervise and instruct patients during the time of WCD prescription. Nevertheless, prospective and randomized trials are needed to show the impact of these telemonitoring features on patients’ outcome and compliance.

### Limitations

This study has several notable limitations due to its retrospective character. Furthermore, it is a single-center and non-randomized study. We can only provide retrospective follow-up data. We only had retrospective access to the WCD supplier’s network. As we only included patients between 2016 and 2020, those patients were not treated according to the 2021 heart failure guidelines but rather to their precursors [24]. This may explain the low percentage of patients with prescribed sarcubitril/valsartan. We cannot provide data on the impact of body angle, body positions or daily steps on the patients’ outcome. Furthermore, we cannot correlate these values with documented arrhythmias. Lastly, we can only provide the NYHA stage and BNP values at admission before any treatment.

## 5. Conclusions

WCDs may be used as a telemonitoring system in patients with HFrEF. Women, patients over the age of 65 and patients who are overweight or obese showed decreased physical activity and may potentially benefit from the short-term intervention of their treating heart-failure expert. Moreover, changes in sleeping angle or position may indicate the worsening of heart failure. Here, further prospective studies should be conducted.

## Figures and Tables

**Table 1:** Baseline characteristics.

	*n* = 140
Sex (female)	32 (22.86%)
Age (years)	57.91 ± 12.76
Diagnosis (ICM/DCM)	ICM: 78 (55.71%) DCM: 62 (44.29%)
First diagnosis	104 (74.28%)
Ejection fraction (%)	26.9 ± 7.35
NYHA stage	2.80 ± 0.86
BMI	29.29 ± 7.04
CAD	92 (65.71%)
ATH	89 (63.57%)
History of stroke	16 (11.43%)
Diabetes mellitus II	38 (27.14%)
OSAS	17 (12.14%)
COPD	19 (13.57%)
History AF	40 (28.57%)
Creatinine (mg/dL)	1.09 ± 0.49
BNP (pg/mL)	4798.34 ± 6769.94

**Table 2:** Medication at discharge.

	*n* = 140
Betablocker	140 (100%)
ACE-I/AT	99 (70.71%)
ATR	127 (90.71%)
ARNI	40 (28.57%)
Ivabradin	11 (7.87%)
Digitalis	13 (9.28%)
OAC	53 (37.85%)

**Table 3:** Follow-up data.

	*n* = 140
EF after follow-up	37.05 ± 9.12
ICD/CRT-D	39 (27.85%)
Compliance (h/d)	21.39 ± 3.98
Duration of WCD use (days)	59.78 ± 35.72
Average heart rate (bpm)	70.06 ± 11.89
Average daily steps	6027.45 ± 3564.38
Average upright position (%)	52.15 ± 15.67
Average reclined position (%)	17.64 ± 10.51
Average flat position (%)	30.15 ± 14.93
Average left position (%)	22.59 ± 14.53
Average prone position (%)	9.31 ± 13.38
Average right position (%)	21.23 ± 14.58
Average supine position (%)	46.91 ± 20.45

**Table 4:** Linear regression analysis; CI = confidence interval.

	Coefficient	95% CI	*p*-Value
**Age (years)**	−59	−111; −7	0.027
**BMI**	−117	−186; −47	0.001
**Sex (m vs. f)**	1347	95; 2599	0.035

## Data Availability

All data are presented in this manuscript.
